# Lancet Commission on Hearing Loss: d/Deaf and Hard of Hearing Stigma Measurement Tools

**DOI:** 10.1093/geroni/igaf122.3410

**Published:** 2025-12-31

**Authors:** Jessica West, Rachel Stelmach, Melissa Stockton, Howard Francis, John Kraemer, Khalida Saalim, Elizabeth Troutman Adams, Laura Nyblade

**Affiliations:** Duke University School of Medicine, Durham, North Carolina, United States; RTI International, Durham, North Carolina, United States; University of Pennsylvania Perelman School of Medicine, Philadelphia, Pennsylvania, United States; Duke University School of Medicine, Durham, North Carolina, United States; Georgetown University, Washington, District of Columbia, United States; RTI International, Durham, North Carolina, United States; RTI International, Durham, North Carolina, United States; RTI International, Durham, North Carolina, United States

## Abstract

The Lancet Commission on Hearing Loss Measures, Models, and Reduction of Stigma Subgroup aims to advance understanding of d/Deaf or hard of hearing (d/DHH) stigma and actions to address it. We identified a lack of tools to measure the different types of stigma faced by d/DHH people and their close associates. Here, we describe the development and preliminary validation of novel d/DHH stigma measurement tools. We used a multistep exploratory sequential design: survey development, modified Delphi process, cognitive interviewing, and pretesting. Psychometric validation occurred in the U.S. (high-income country) and Ghana (low-middle income country). Stigma measurement tools were developed for 1) d/DHH individuals (experienced, perceived, internalized, anticipated hearing device-related stigma [HDRS]); 2) care partners of d/DHH adults (observed and perceived stigma toward d/DHH people; care partners’ internalized, experienced, perceived stigma; perceived HDRS); 3) parents of d/DHH children (parental observations and perceptions of stigma; parental secondary experienced, perceived, internalized stigma; perceived HDRS); and 4) health care providers (HCPs) (perceived and enacted stigma; secondhand patient-experienced stigma; secondhand patient-perceived stigma; perceived HDRS). Additional ageism scales measure d/DHH peoples’ experienced ageism and observed ageism among care partners, HCPs, and the general population. These tools, available in Ear and Hearing, fill a critical gap in d/DHH stigma measurement. Ongoing research using these tools will enable monitoring of d/DHH stigma, refinement of interventions, and assessment of societal attitudes toward d/DHH people. Leveraging these tools in clinical and public health settings can help reduce stigma, improve patient experiences, and lead to better overall outcomes in hearing healthcare.

